# Dosimetric analysis of the brachial plexus among patients with breast cancer treated with post-mastectomy radiotherapy to the ipsilateral supraclavicular area: report of 3 cases of radiation-induced brachial plexus neuropathy

**DOI:** 10.1186/s13014-014-0292-5

**Published:** 2014-12-12

**Authors:** San-Gang Wu, Si-Juan Huang, Juan Zhou, Jia-Yuan Sun, Han Guo, Feng-Yan Li, Qin Lin, Huan-Xin Lin, Zhen-Yu He

**Affiliations:** Department of Radiation Oncology, Xiamen Cancer Center, the First Affiliated Hospital of Xiamen University, Xiamen, 361003 People’s Republic of China; Department of Radiation Oncology, Collaborative Innovation Center of Cancer Medicine, State Key Laboratory of Oncology in South China, Sun Yat-sen University Cancer Center, Guangzhou, 510060 People’s Republic of China; Department of Obstetrics and Gynecology, Xiamen Cancer Center, the First Affiliated Hospital of Xiamen University, Xiamen, 361003 People’s Republic of China; Department of Basic Medical Science, Medical College, Xiamen University, Xiamen, 361003 People’s Republic of China

**Keywords:** Breast cancer, Radiation therapy, Brachial plexus, Radiation-induced brachial plexus neuropathy

## Abstract

**Background:**

The purpose of this study was to evaluate the brachial plexus (BP) dose of postmastectomy radiotherapy (PMRT) to the ipsilateral supraclavicular (ISCL) area, and report the characteristics of radiation-induced brachial plexus neuropathy (RIBPN).

**Methods:**

The BP dose of 31 patients who received adjuvant PMRT to the ISCL area and chest wall using three-dimensional conformal radiotherapy (3DCRT) and the records of 3 patients with RIBPN were retrospectively analyzed based on the standardized Radiation Therapy Oncology Group-endorsed guidelines. The total dose to the ISCL area and chest wall was 50 Gy in 25 fractions.

**Results:**

Patients with a higher number of removed lymph nodes (RLNs) had a higher risk of RIBPN (hazard ratio [HR]: 1.189, 95% confidence interval [CI]: 1.005-1.406, p = 0.044). In 31 patients treated with 3DCRT, the mean dose to the BP without irradiation to the ISCL area was significantly less than that with irradiation to the ISCL area (0.97 ± 0.20 vs. 44.39 ± 4.13 Gy, *t* = 136.75, p <0.001). In the 3DCRT plans with irradiation to the ISCL area and chest wall, the maximum dose to the BP was negatively correlated with age (*r* = −0.40, p = 0.026), body mass index (BMI) (*r* = −0.44, p = 0.014), and body weight (*r* = −0.45, p = 0.011). Symptoms of the 3 patients with RIBPN occurred 37–65 months after radiotherapy, and included progressive upper extremity numbness, pain, and motor disturbance. After treatment, 1 patient was stable, and the other 2 patients’ symptoms worsened.

**Conclusions:**

The incidence of RIBPN was higher in patients with a higher number of RLNs after PMRT. The dose to the BP is primarily from irradiation of the ISCL area, and is higher in slim and young patients. Prevention should be the main focus of managing RIBPN, and the BP should be considered an organ-at-risk when designing a radiotherapy plan for the ISCL area.

## Background

Clinical trials have confirmed that adjuvant postmastectomy radiotherapy (PMRT) can improve the locoregional control rate (LCR) and survival rate of patients with locally advanced breast cancer, especially with axillary lymph node metastasis [1–3]. The incidence of recurrence in ipsilateral supraclavicular or infraclavicular fossa without PMRT is 4%-8%, when patients had locoregional recurrence, supraclavicular or infraclavicular fossa was involved in 23%-43% of failures [4–7]. Therefore, the guidelines of the National Comprehensive Cancer Network (NCCN) and the German Society of Radiation Oncology (DEGRO) recommend irradiation to chest wall (breast) and the ipsilateral supraclavicular (ISCL) area for patients with locally advanced breast cancer [8,9]. Because of the curative intent of breast cancer surgery, attention should be given to late-stage injury after PMRT. It has been reported 14%-20% breast cancer patients developed radiation-induced brachial plexus neuropathy (RIBPN) after radiotherapy in recent years [10,11].

The brachial plexus (BP) is formed by the last 4 cervical nerves (C5-C8) and the 1st thoracic nerve (T1). RIBPN symptoms include upper extremity numbness, pain, weakness, and motor disturbance. RIBPN is slowly progressive and often leads to permanent disability and seriously affects the quality of life.

The risk of RIBPN is interested in head and neck cancer treated with high-dose radiation therapy and lung cancer treated with stereotactic body radiotherapy [12,13]. However, because the conventional fractionated irradiation method is more commonly used in PMRT, BP injury should be different from that occurring with stereotactic body radiation therapy. At present, the BP is not always regarded as an organ-at-risk (OAR) in the optimization and restriction of plans designed for the radiotherapy of breast cancer.

Thus, the purpose of this study was to perform a retrospective analysis of the radiation dose to the BP in 3DCRT plans with PMRT to the ISCL area and chest wall. We further investigated the dosimetric features of the BP in radiotherapy, and the potential correlation with physical features. Lastly, we reported 3 patients with RIBPN after PMRT.

## Materials and methods

### Patients

We retrospectively analyzed the 3DCRT plans of 31 breast cancer patients who received PMRT to the ISCL area and chest wall between January 2007 and December 2007 at Sun Yat-sen University Cancer Center (Group 1). In addition, we reviewed the records of 3 patients with RIBPN who were admitted to our center between January 2001 and December 2007 (Group 2). RIBPN can affect both sensory and motor function of the ipsilateral arm and hand, i.e. paraesthesia, oedema, pain, and dyskinesia. In present study, RIBPN was graded using a modified late effects of normal tissue-subjective, objective, management, and analytic (LENT-SOMA) score [14–16]. The study was approved by the ethics committee of Sun Yat-Sen University Cancer Center. All patients provided written consent for storage of their medical information in the hospital database and for research use of this information.

### Contouring of the brachial plexus

**Figure 1:**
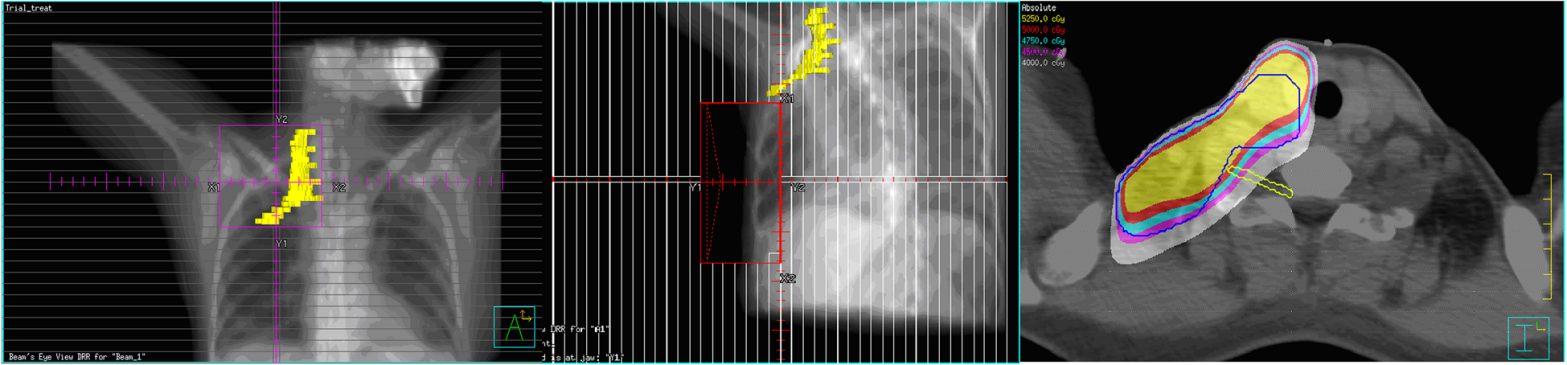
**Beam eye view (BEV) and cross-sectional diagram of the brachial plexus (PTV marked with blue lines).**

In both groups the BP was contoured according to the standardized Radiation Therapy Oncology Group (RTOG)–endorsed guidelines delineation [17]: 1) Identify and contour C5, T1, and T2; 2) Identify and contour the subclavian and axillary neurovascular bundle; 3) Identify and contour anterior and middle scalene muscles from C5 to insertion onto the first rib; 4) Contour the brachial plexus as an organ at risk (OAR) using a 5-mm diameter paint tool; 5) Start at the neural foramina from C5 to T1; this should extend from the lateral aspect of the spinal canal to the small space between the anterior and middle scalene muscles; 6) For computed tomography (CT) slices, where no neural foramen is present, contour only the space between the anterior and middle scalene muscles; 7) Continue to contour the space between the anterior and middle scalene muscles; eventually the middle scalene will end in the region of the subclavian neurovascular bundle; 8) Contour the brachial plexus as the posterior aspect of the neurovascular bundle inferiorly and laterally to 1–2 CT slices below the clavicular head; 9) The first and second ribs serve as the medial boundary of the OAR contour (Figure 1).

### Treatment plan

All patients both groups were irradiated to the ISCL area and chest wall (CW). The total dose was 50 Gy in 25 fractions. The patient had a supine treatment position. All patients had three-dimensional dose planning (Pinnacle 7.4f ®; Philips Medical Systems, Bothell, WA, USA) based on CT scanning with a slice thickness of 5 mm. A planning target volume (PTV) including the chest wall, supraclavicular and infraclavicular (SIF) fossa as delineated. A common isocentre for all fields independent of the technique was located in the junction between the SIF fields and CW fields. A high-energy linac with photon beams and electron beams was used. All treatment plans were individually optimized. The ISCL field dose was specified to cover > 95% PTV with at least 95% of the prescribed dose. The ISCL was irradiated with 6 MV X-ray and 12–15 MeV electron beam, and the CW was irradiated with a 6 MV X-ray tangent field. The axillary and internal mammary lymph nodes were not irradiated. Thirty-one patients were redesigned the treatment plan without irradiation to ISCL to compare the radiation doses to the BP with irradiation to the ISCL in Group 1 using dose-volume histograms (DVH). The correlation between patient clinical features and the radiation dose to the BP with irradiation to the ISCL area and CW was determined. Three patients with RIBPN after PMRT also were examined with respect to the radiation dose to the BP and clinical features.

### Statistical analysis

SPSS 19.0 software was used for data analysis. The correlation between the radiation dose to the BP and clinical features was analyzed using Pearson’s correlation coefficient. The independent effects of the clinical and dosimetric parameters associated with RIBPN development were determined by univariate and multivariate logistic regression analysis. Factors with statistically significant differences in univariate analysis were included in multiple logistic regression analysis. The correlation between the radiation dose to the BP and clinical features was analyzed using Pearson’s correlation coefficient. Radiation doses of various plans were compared using paired *t* test. A value of p < 0.05 was considered to be statistically significant.

## Results

### The correlation of clinical and dosimetric parameters with RIBPN

Table 1 shows the correlation of clinical and dosimetric parameters with RIBPN using univariate logistic regression analysis. Patients with a higher number of RLNs had a higher risk of RIBPN (hazard ratio [HR]: 1.189, 95% confidence interval [CI]: 1.005-1.406, p = 0.044). Patients with younger age (p = 0.056), shorter height (p = 0.073) and lower weight (p = 0.090) had a borderline significant trend with RIBPN development. The further multivariate logistic regression analysis was not performed due to only one factor had predictive value with RIBPN development.

**Table 1:** **The correlation of clinical and dosimetric parameters with RIBPN**

**Characteristic**	**Group 1**	**Group 2**	**p**
Age (y)	49 (34–63)	36 (31–43)	0.056
Height (m)	1.58 ± 0.05	1.51 ± 0.03	0.073
Weight (kg)	56.13 ± 7.38	47.60 ± 1.83	0.090
BMI (kg/m^2^)	22.52 ± 2.44	20.86 ± 0.28	0.259
Number of RLNs (n)	23.42 ± 6.51	34.00 ± 10.15	0.044
Dmean (Gy)	44.39 ± 4.13	47.03 ± 1.54	0.286
Dmax (Gy)	56.16 ± 2.58	59.33 ± 0.83	0.111
V40 (%)	79.90 ± 10.74	87.55 ± 2.91	0.202
V45 (%)	68.22 ± 12.63	76.29 ± 3.85	0.271
V50 (%)	49.25 ± 16.58	58.97 ± 3.41	0.320
V52.5 (%)	28.52 ± 18.98	32.12 ± 3.70	0.740
V55 (%)	6.08 ± 10.36	5.81 ± 1.51	0.963

BMI: body mass index; RLNs: removed lymph nodes.

Vn indicates the volume of the BP receiving n Gy.

### BP dose, equivalent uniform dose, and body mass index of Group 1

**Figure 2:**
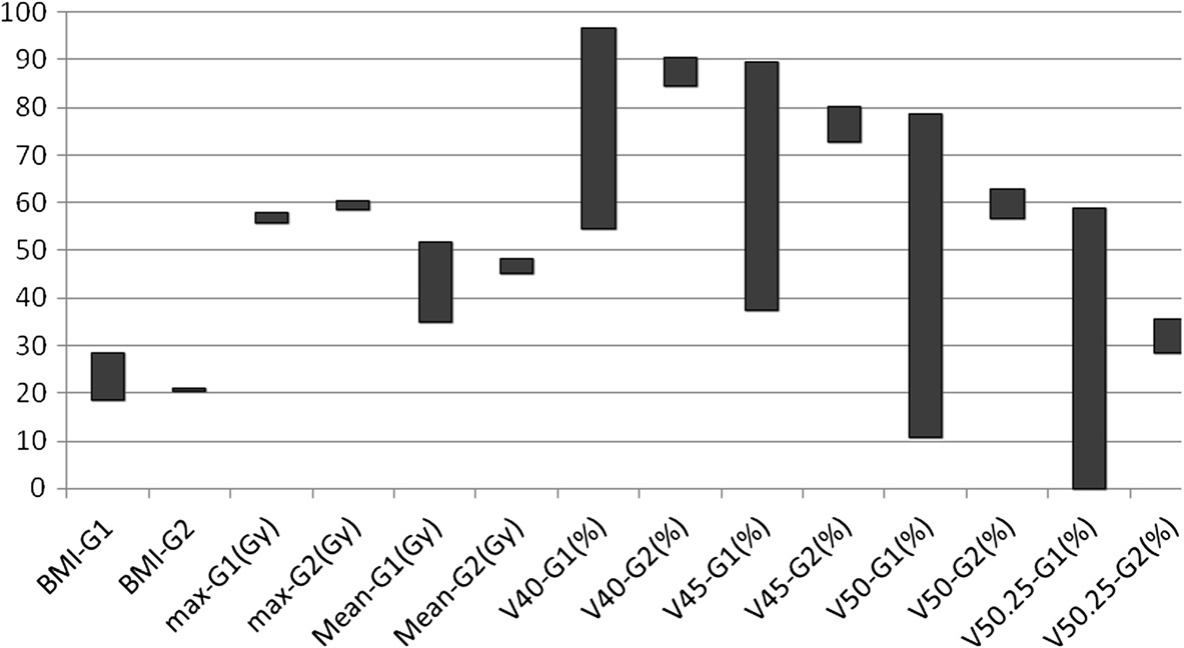
**Clinical data and radiation dose to the brachial plexus in group 1 and group 2 patients.**

The median follow-up time of 31 patients was 80.0 months (range, 74–85 months). The mean dose (Dmean) to the BP without irradiation to the ISCL area was 0.97 ± 0.20 Gy, and the dose to the BP increased significantly with irradiation to the ISCL area (*t* = 136.75, p < 0.001) (Table 1 and Figure 2). In the 3DCRT plans with irradiation to the ISCL area and chest wall, the maximum dose (Dmax) to the BP was negatively correlated with age (*r* = −0.40, p = 0.026), body mass index (BMI) (*r* = −0.44, p = 0.014) and body weight (*r* = −0.450, p = 0.011). There was no correlation between BP Dmax and height, and there was no correlation between BP Dmean and age (*r* = −0.18, p = 0.340), height (*r* = −0.01, p = 0.990), BMI (*r* = −0.34, p = 0.060), or weight (*r* = −0.28, p = 0.120).

### BP dose and clinical characteristics of patients with RIBPN (Group 2)

Between January 2001 and December 2007, a total of 629 breast cancer patients whose median follow-up duration was 63.1 months (range, 6–130 months). The postmastectomy CW and ISCL area received a dose of 50 Gy in 25 fractions with 3DCRT. All patients received 3DCRT after chemotherapy, and none of these patients received concurrent chemoradiotherapy. Of these patients, 3 (0.48%) developed RIBPN. Symptoms of RIBPN in the 3 patients occurred in a median of 39 months (range, 37–65 months) after radiotherapy. The time interval between the first appearance of symptoms and diagnosis was more than 6 months in all patients. Symptoms of the affected limbs were aggravated in all 3 patients, and progressive neuropathic pain occurred. Two patients had decreased muscle power of the intrinsic muscles and flexion dysfunction. Physical examination showed that there were no significant differences between the healthy limb and the affected limb in skin color and temperature. Only enlargement of the limb circumference because of lymphedema was noted. Electromyography (EMG) showed injury of the BP trunk. Tumor factors were excluded by magnetic resonance imaging (MRI). Symptomatic treatments such as analgesics and neurotrophic drugs were administered to all 3 patients, and 2 patients also received physical rehabilitation. After treatment, 1 patient was stable, and the other 2 patient’s symptoms worsened. The dose to the BP and clinical characteristics of the patients with RIBPN are shown in Tables 1 and 2 and Figure 2.

**Table 2:** **Characteristics of patients with radiation-induced brachial plexus neuropathy**

**Characteristic**	**Patient 1**	**Patient 2**	**Patient 3**
Tumor location	Left breast	Left breast	Right breast
Staging of the primary tumor (AJCC 2008)	T2N2M0	T1N3M0	T3N3M0
Surgeries	MRM and ALND	MRM and ALND	MRM and ALND
Chemotherapy	6 courses of DCE	6 courses of CEF	6 courses of CEF
Endocrine therapy	-	TAM	TAM
Onset of symptoms after radiotherapy	35 months	27 months	29 months
Symptom occurrence to seeking care	7 months	12 months	10 months
Initial symptoms	Sensory disorders	Motor deficits	Motor deficits
Overall symptoms	Pain, sensory disorders	Motor deficits, pain, sensory disorders	Motor deficits, pain, sensory disorders
Localization of injury	C5-Th1	C5-7	C5-Th1
Severity of injury (LENT-SOMA scale)	Grade 2	Grade 2	Grade 3
Upper-extremity edema	Yes	No	No
Time of treatment	27 months	31 months	12 months
Treatment outcome	Stable	Slow progression	Slow progression

MRM: modified radical mastectomy; ALND: axillary lymph node dissection; DCE: docetaxel, cyclophosphomide, epirubicin; CEF: cyclophosphamide, epirubicin, 5-fluorouracil; LENT-SOMA: late effects of normal tissue-subjective, objective, management, and analytic.

## Discussion

Our analyses of the 3DCRT plans of 31 patients who received PMRT to the ISCL area and CW showed that the dose to the BP was related to irradiation to the ISCL area. This finding is consistent with that of Stanic et al. [18]. There have been few studies on the occurrence of RIBPN symptoms, including worsening chronic pain and decreased sensory and motor function, among the patients with breast cancer, head and neck cancer, and lung cancer after irradiation to the BP [10–13,19]. Importantly, at present RIBPN is an incurable complication.

There is a significant difference in the incidence of RIBPN in the literature. RIBPN incidence is in accordance with the irradiation technique, and ranges from 66% RIBPN with 60 Gy in 5 Gy fractions in the 1960s to less than 1% with 50 Gy in 2.0 Gy fractions today [19]. In present study, the incidence of RIBPN was 0.48%. However, a study which 20% patients developed RIBPN when irradiation to the supraclavicular lymph nodes and chest wall (breast) using 3DCRT technique with 50 Gy in 2.0 Gy fractions [11]. Based on the radiation technique in modern era, the combined treatment-related factors (surgery in the case of haematoma or chronic infection and extended axillary lymph node dissection, *et al*.) and the patient-related factors (young or advanced age, obesity, hypersensitive patients, or smoking, *et al.*) may affect the risk, severity, and nature of RIBPN [20]. The follow-up time for different study may also affect the incidence of RIBPN.

It has been reported that the incidence of RIBPN is primarily related to the total dose and fractionated dose to the BP [21–23]. Moreover, Killer et al. reported the incidence of RIBPN was positively correlated with the dose to the BP [24]. These findings may be related to the fact that the BP is a serial organ. Therefore, Emami et al. [25] have suggested that the dose tolerance for a 5% risk of developing RIBPN at 5 years is 62, 61 and 60 Gy, and for a 50% risk at 5 years the dose tolerances are 77, 76 and 75 Gy for one-third, two-thirds and the whole organ respectively. Lundstedt et al. reported that the incidence of RIBPN is 20% after conventional fractionation radiotherapy when the prescribed dose to ISCL is 50 Gy [11].

Though the prescribed dose in our patients was 50 Gy, the BP Dmax was higher than 110% of the prescribed dose in some cases. Moreover, for patients with lymph node metastases in the ISCL area, the local dose with boost could be up to 60–70 Gy, which may lead to an increased incidence of RIBPN [26]. For these reasons, we suggest that the BP should be considered as one of the OAR when the ISCL area is planned to be irradiated. But we can not come up with a recommendation of the appropriate dose constraints to brachial plexus due to limited number of patients. The Danish Breast Cancer Cooperative Group recommend the maximum dose to BP should not exceed 54 Gy [27]. In present study, the Dmax to BP in Group 1 and Group 2 are more than 54 Gy. A French study suggested that the Dmax to BP should not exceed 60 Gy, even if possible, 50 Gy [28]. Conventionally, ISCL fields are matched on to tangential breast fields using various techniques [29], the difficulties in matching treatment fields to achieve homogenous dose distribution may results in overdose to BP. Therefore, it is very difficult to achieve a dose of less than 50 Gy of BP. Helical tomotherapy and integrated IMRT treatment plans improved the dose distribution of the supraclavicular region and showed better dose conformity and uniformity of the integrated target volume of the chest wall and supraclavicular region without the requirement of field matching [26,30]. Thus, the new irradiation techniques with brachial plexus-sparing may be beneficial to the protection of the brachial plexus.

There have few studies on the relationship between the dose to the BP and morbidity, disease severity, and chemotherapy [24]. In addition, unconventional fractionated irradiation is becoming more widely used, which may potentially lead to an increased incidence of RIBPN [10,21,31]. Thus, it is necessary to establish risk models for RIBPN based on DVH or normal tissue complication probability models in further research.

In present study, the Dmax to BP was not associated with RIBPN, but patients with a higher number of RLNs were the independent factor associated with RIBPN development. Thus, the dose to the BP should be decreased as much as possible when the dose to the planned target volume is satisfied in patients with a higher number of removed lymph nodes. In addition, lymphedema after axillary dissection can cause brachial plexus neuropathy in breast cancer patients. It was about 13% patients developed brachial plexus neuropathy after axillary dissection without regional radiotherapy. Radiotherapy to the supraclavicular lymph nodes after axillary dissection increases the incidence of brachial plexus neuropathy. When adjusted for lymphedema the contribution from radiotherapy is no longer formally statistically significant indicating that there is also an indirect effect mediated by the lymphedema [11].

Olson et al. [32] and Lundstedt et al. [11] have reported that the incidence of RIBPN was higher in young patients. This is consistent with our finding of a negative correlation between BP Dmax and age. However, it was also reported that there was no correlation between age and the incidence of RIBPN [23]. In addition, our study showed that BP Dmax was negatively correlated with BMI and body weight, which is the same as reported by Klein et al. [33]. These findings indicate that the dose to the BP is relatively higher in slim patients. This, to a certain degree, explains why RIBPN is more likely to occur in slim and young patients. In present study, patients with younger age, shorter height and lower weight had a borderline significant trend with RIBPN development. The further studies with a larger sample size are needed to confirm our study.

Of the 629 patients with irradiation to the ISCL area and chest wall, only 3 (0.48%) developed RIBPN. The development of the RIBPN symptoms in the 3 patients had a relatively long latent period; thus, a diagnosis of RIBPN may be delayed because of mile symptoms in the early stage of the condition. On the other hand, the disease results in progressive and irreversibility deterioration and conservative treatment is ineffective. Surgical treatments including BP neurolysis and revascularization by enveloping the BP using a greater free omental flap have not provided satisfactory results [23]. Induced pluripotent stem cell (IPSC) therapy is a new promising technique that is still in the preclinical testing stage. Because comprehensive management of breast cancer has resulted in greater long-term survival, greater attention should be paid to prevent the occurrence of RIBPN.

There are several limitations to this study that should be considered. First, the study was retrospective, and the sample size was relatively small. In addition, the follow-up duration was relatively short, and the number of reported RIBPN cases was small. Therefore, the results cannot represent the majority of population.

## Conclusions

In summary, the incidence of RIBPN was higher in patients with a higher number of RLNs after PMRT. The dose to the BP is primarily from irradiation to the ISCL area, and is higher in slim and young patients. Prevention should be the main focus of managing RIBPN, especially for slim and younger patients. Based on the results of this study, we suggest that the BP should be considered an OAR when designing a radiotherapy plan for the ISCL area.

## Abbreviations

BP: Brachial plexus
PMRT: Post-mastectomy radiotherapy
ISCL: Ipsilateral supraclavicular area
RIBPN: Radiation-induced brachial plexus neuropathy
3DCRT: Three-dimensional conformal radiotherapy
BMI: Body mass index
LCR: Locoregional control rate
RLNs: Removed lymph nodes
HR: Hazard ratio
CI: Confidence interval
PTV: Planning target volume
CW: Chest wall
SIF: Supraclavicular and infraclavicular
LENT-SOMA: Late effects of normal tissue-subjective, objective, management, and analytic
NCCN: National comprehensive cancer network
DEGRO: German society of radiation oncology
IMRT: Intensity-modulated radiation therapy
OAR: Organ at risk
RTOG: Radiation therapy oncology group
CT: Computed tomography
DVH: Dose-volume histograms
EMG: Electromyography
MRI: Magnetic resonance imaging
IPSC: Induced pluripotent stem cell
